# Some Account of Epidemic Erysipelas as It Prevailed in Some Parts of Ohio

**Published:** 1845-08

**Authors:** John Dawson

**Affiliations:** Jamestown, Ohio


### WESTERN JOURNAL

OF

### MEDICINE AND SURGERY.

AUGUST, 1845.

### Art. I.—*An account of Epidemic Erysipelas as it prevailed in some parts of Ohio.* By John Dawson, M.D., of Jamestown, Ohio.

Epidemic Erysipelas having prevailed for some time past in various localities of the Mississippi Valley, including our own district, Green county, Ohio, I concluded to spend a portion of my time in visiting several places, reputed to have suffered no little from its malignant effects. Before attempting to give a description of the malady as it manifested itself during its late epidemic prevalence, it may not be amiss to premise a few remarks on what is usually denominated *spasmodic* erysipelas.

Numerous have been the divisions which have obtained among medical and chirurgical writers. Swan divided the disease into *idiopathic* and *symptomatic;* Willan, into *erysipelas phlegmonodes*, *e. edematodes*, *e. gangrenosum*, and *e. erra-*

*ticum;* Dessault, into *phlegmonous*, *bilious*, and *local;* Pearson, into *phlegmonous*, *œdematous*, and *gangrenous;* Lawrence, into *erythema simple*, *œdematous*, and *phlegmonous;* Burserius, into *idiopathic*, or that which arises from spontaneous or internal causes, not preceded by any other disease, *symptomatic*, depending upon another disease by which its progress is completely influenced, and *accidental*, or that which is casually excited by some external manifest cause; Good into *local* and *erratic.* Abernethy supposed it was always *symptomatic;* hence he says, "I'll be hanged if erysipelas is not always the result of a disordered state of the digestive organs." Cullen makes no division at all, but considers the malady as having little or no relation to erythema. I might refer to other authors, ancient and modern, on the divisions under which it would be most convenient to discuss the various lesions, functional and structural, displayed by erysipelas, but have referred to enough for my purpose; and shall, without expressing an opinion on the merits or demerits of any of the divisions to which I have alluded, proceed to speak of the disease under three heads, the *simple*, *œdematous*, and *phlegmonous.*

That form of the malady of most frequent occurrence is the one in which there appears on some portion or other of the body, a small patch of vesications seated upon an inflamed skin, and exhibiting but little tendency to spread. This in general, makes its appearance without our being able to attribute it to any specific cause, and in a great proportion of cases is unaccompanied with any febrile excitement. By some of the older writers it is denominated "*rose*," by others "*blight.*" A large proportion of all the cases entitled sporadic erysipelas which I have witnessed, for the last six or seven years, has been of this description. So far as I can now recollect, its most frequent seat has been upon the abdomen and chest; and in a few instances on the extremities. Treated locally, this affection, in my practice, has pretty uniformly subsided. Once in a while I believe it does happen that by extending itself to a considerable portion of the surface a source of irritation is established, the consequences of which, sooner or later, are felt in the circulation. Such issues, however, are exceedingly rare.

Grave and much more serious is that form of erysipelas where the dermoid tissue seems to be the seat of a burning pain, a high degree of inflammation, and considerable tumefaction. Here we have fever of the synochus grade, and more or less disturbance of the secretions. The eruption in this variety exhibits a great tendency to spread. Occurring as is commonly the case, first upon the neck or face, it travels to the head, breast, shoulder, or superior extremities. Usually it does not extend much below the dermoid tissue, but seems to spend the principal force of its morbid actions in producing inflammation of the skin itself, and the effusion of serum between its laminæ. From the fact that the effusion of serum gives to the skin a swelled doughy appearance, this variety, by pretty general consent, has been denominated the *œdematous.*

A form of the disease differing in no very essential respect from the one just described, except in severity and the extent of the parts involved, is that in which not only the skin but also the subcutaneous and inter-muscular cellular tissues are the parts involved. Being the most interesting form of the malady I will notice the manner in which it invades the system; and some of the pathological habitudes by which it is characterized. Usually the attack is preceded by rigors, pain in the back, head, and extremities. Added to these, in a short time, there is fever, which in persons of a full habit is apt to be of synochus grade, but in those where the habit is bad from constitutional debility or predisposition to disease, the reaction in the vascular system will be found to be typhoid. Sooner or later the local disease makes its appearance on the face, neck, head, or extremities. Of a bright red color at first, the parts, in some cases, assume a livid hue, and once in a while are slightly yellow. On pressure the color disappears, but it is again assumed when the pressure is discontinued. The lesions of sensibility consist in a burning pain, at times attended with intolerable itching. A tumor more or less extensive makes its appearance, upon which vesicles are situated, filled at first with a clear fluid, which becomes yellow. After a few days these vesicles burst, take on the form of cancerous ulcers, which rapidly burrow through the integuments and communicate their morbid action to the parts beneath the skin, the subcutaneous cellular tissue, and fascia covering the muscles. Formed once in the cellular tissue beneath the skin, and that enveloping the muscles, the process of suppuration becomes protracted, giving rise to sinuses, subcutaneous abscesses, and purulent depots. From the tendency of this variety to those puriform infiltrations and subcutaneous abscesses, it is very appropriately denominated the *phlegmonous* variety of erysipelas. Obviously different is it however from that kind of diseased action to which the name phlegmon has been applied. In common phlegmon the matter formed is generally healthy pus; in erysipelas it is bloody serum; in phlegmon the matter is in a circumscribed cavity; in erysipelas it is effused under all the parts of the skin diseased, following the course of the cellular tissue and fascia covering the muscles.

Without referring to the *erysipelas phlegmonodes biliosum* of the Continental and some American writers, or to the *duratia telæ cellularis*, the former of which is a mere complication of erysipelas with hepatic disease, and the latter peculiar to infants, and in this country exceedingly rare, we shall proceed to notice *epidemic erysipelas*, that we may see what relation it bears to the different varieties of the disease we have been describing.

### HISTORY OF EPIDEMIC ERYSIPELAS.

New as the disease is to most of the practitioners of the present day, it can nevertheless boast of considerable antiquity. Hippocrates mentions an erysipelas which spread among the people and proved very fatal, affecting an arm, leg, &c., with gangrenous ulceration. Galen and Celsus speak of the malady, though in terms less definite. The first regular account of the disorder was published by the Medical Professors at Marpurgh in the year 1597 (Good). Epidemic erysipelas or something very much like it was observed by Dr. Patrick Russell, physician to the British factory at Aleppo in 1660, 1661 and 1662. In 1665 Sydenham says that an erysipelas, or *ignis sacer* prevailed in connexion with the plague.—Swan gives an account of erysipelas as having prevailed with phenomena very much like plague. De Haen and Bertholini mention epidemic erysipelas, and we also have accounts of its prevalence at Toulouse in 1716, attended with great mortality. Amply sufficient are these references to show, that epidemic erysipelas, instead of being a modern malady is one that has been known for centuries. From the hospital reports of this country and Europe we find that these institutions have suffered with more or less violence at various periods. In the Transactions of the College of Physicians of Philadelphia, 1842, there is a statement from Dr. Stewardson, resident physician of the Pennsylvania Hospital, that epidemic erysipelas was in the surgical ward of that institution during the year of 1830. Dr. Condie stated that during the year previous to the time the college was in session, epidemic erysipelas had prevailed in the southern districts of the city of Philadelphia. Dr. E. McDowell, one of the surgeons of the Richmond Hospital gives an account of the disease as being epidemic in the Dublin Hospitals in 1834. At about the same period, as well as afterwards, the disease was epidemic in western New York, and also to a slight extent in the Mississippi Valley. In the Western Journal of the Medical and Physical Sciences, Dr. Jesse Paramore, formerly of Eaton, Ohio, gave a brief account of the disease which he says was epidemic in his county (Preble) during the winter of 1835–'6. There is but little doubt that the erysipelas of '35–'6 was in many respects similar to that with which we have recently been visited. The accounts, however, are so brief and imperfect, that we can tell but little of the various morbid phases which it assumed. The first intimation we had of its late prevalence was public rumor. It went, as upon the wings of the wind, that a disease called the "*black-tongue*" was making fearful ravages in various localities of the West and South, and in due time the rumor was to some extent confirmed by a communication in the Western Lancet, from the pen of Dr. Sutton of Indiana. During the winter and spring of '44 the disease assumed the form of an epidemic in the Miami Valley. At first it was most severly felt in the vicinity of the larger streams of water. On each side of the Miamis, and along some of their principal tributaries it made its first appearance, shewing as it were a preference for malarious districts. The inhabitants of table lands near these streams, as well as the flat regions in which they take their rise, although not exempt, were nevertheless not so generally affected; and in such localities, from what we have learned, the disorder has been more mild. Dayton, situated at the confluence of Mad River and the Big Miami suffered as severely as perhaps any other point in Ohio. Centreville, nine miles distant, was also severely visited. In our county (Green) the malady was met with at various points during the past year, and was in the general characterized by no uncommon violence.

As far as could be ascertained all ages contracted the disorder, though not perhaps with the same facility. Some have thought that young children were more exempt than any other class. To this opinion I feel strongly inclined, though we saw one case which took place in a child aged 11 months. In his account of the disease as published in the November number of the Western Lancet, Dr. Sutton says that he witnessed no case in a subject under two years of age.

Some physicians, with whom we have conversed, think females most predisposed. In part, this opinion is due to the fact that wherever erysipelas has prevailed with much mortality, females have been the greatest sufferers. Without doubt the greatest mortality has been among females; but it is not quite so certain that the greatest number of them have been affected with the malady.

Our acquaintance with the disease has not been sufficient to ascertain the habit upon which it is most likely to fall. It has been thought that it exhibited a preference for the scrofulous or phthisical, or that vitiated by intemperance. Certain it is that those of bad habit had the disease with the greatest severity, but all classes, those of good constitution, as well as the infirm, had contracted the malady pretty much alike.

Erysipelas has appeared in our county under various grades of violence. Sometimes it has been comparatively mild, at other times extremely severe and dangerous. On this account, therefore, in describing it I shall be compelled to divide it into several varieties, founded upon the violence and diversity of the morbid phenomena.

*Of the mild variety.*—Invariably I believe the manner in which the remote cause invaded the system in this, as well as in the other varieties, showed that it produced a general disease, not confined at first to any particular part, but invading the entire organism. Aching of the bones generally, pain in the back and head, chills and flashes of heat, are among the first symptoms of indisposition. To these, fever, commonly of the synochus grade, succeeded. The pulse was usually frequent and full, and very much under the influence of the lancet. Nearly in every case the throat was sore, varying from a mere feeling of uneasiness to a sense of rawness, attended with considerable pain. In most instances the thirst was urgent, and the tongue coated. The skin was sometimes dry, at other times copiously bathed in perspiration. The smaller lymphatic glands about the neck, in most instances, were slightly enlarged, but *there was no appearance of erysipelatous inflammation on the skin.* This form of the disease was by far the most common. Nine out of ten, during the time the epidemic prevailed, were not affected with any other symptoms than those just sketched. Indeed, in some neighborhoods, where from fifty to sixty were affected, no case of a malignant character made its appearance. From four to seven days was the time which the disease required to run its course, and it was in all cases, I believe, attended with a favorable termination. Many cases had no treatment at all, except some domestic applications to the throat, or perhaps a purgative to the bowels; yet notwithstanding this they did well. A number of persons, in truth, were affected with this variety, that supposed they had nothing but a bad cold or a slight attack of angina.

We will now give the notes of a case of the simple or mild variety of the complaint, and as I have had the disease myself in this form, the notes of my own case will be submitted.

I had been attending two young men for several days previous to my own illness, but felt not at all indisposed, until on the morning of the 14th of April. I arose from bed with soreness of throat, thirst, pain in the head, general aching of the bones, with a slight sense of chilliness. I however visited several patients in the village, and one at some distance in the country during the day. In the evening I had fever, and the pulse was 92 to the minute. After the fever was developed, ablutions of cold water were applied to the body until a sensation of chilliness was produced, and I also took a cathartic. On the 15th, the aching of the bones had partially subsided, the throat, however, was very sore, with headache, thirst, and fever. There was no change on the 16th, but on the 17th the lymphatic glands on the anterior and lateral aspects of the neck were swollen and painful; the throat was very sore on the inside, and the lateral half arches, the uvula, tonsils, and parts contiguous were inflamed; but as yet there was no abrasions of the mucous surface in the fauces, nor was there any false membrane secreted. My headache being violent, I took a large hydragogue cathartic, and used a gargle of tincture capsicum and dilute muriatic acid. The cathartic operated well, and by the 18th I was convalescent.

This is a pretty fair specimen of the mild variety of the disease. In some the vascular excitement is much greater than in my case; in others it is not so great. In a few cases which I saw, the determination was sufficient to produce delirium; in my own case it produced violent throbbing cephalalgia. With the majority the constitutional disturbance seemed to be about equal to what takes place in an ordinary case of epidemic influenza.

*Of the malignant variety.*—There has been more or less of this in every locality in which the affection has prevailed. The largest proportion, however, of such cases we think have originated in the malarious districts; and more on the first appearance of the malady than subsequently. Like the mild variety, these cases were seized at first with languor, aching of the bones, pain in the head and back, soreness of throat, etc. To these symptoms there succeeded more or less febrile excitement, and in most cases *an eruption upon the skin.* The seat and character of the eruption were various. It has been known to commence upon the toes, and also upon the ends of the fingers, or if there happened to be an abrasion of the skin on any part of the body this was the point usually selected. When there was nothing present to predispose one part more than another, it selected the side of the head, the neck, the nose, face or ears, as the first place of attack. Once developed it exhibited nothing like uniformity in appearance. In one case it would be confined to a particular part, and present the ordinary vesicular appearance, while in another it would be a mere erythematous blush, appearing and disappearing several times during the progress of the malady. I witnessed the case of a child in which the eruption consisted of small chrystalline vesicles, resembling very small drops of dew. In this case the eruption commenced on the side of the neck, and gradually extended over the whole body and extremities. It had not the inflammatory appearance which we usually see in bullate eruptions, but seemed to consist of very minute elevations of the cuticle filled with a glistening fluid. In another case the first appearance of the eruption was manifested by a redness of the left ear, on which was developed a large blister, similar to that produced by cantharides. From the ear it extended over the side of the head and face, producing a high degree of inflammatory action. In other cases the eruption commenced as in common œdematous or phlegmonous erysipelas, and rapidly extended to contiguous parts, producing great pain, swelling, and suppuration of the dermoid tissue. Some cases have been related to me by Dr. Vantuyl, of Dayton, in which the skin presented a normal appearance until after death, when large red patches of erythema were developed over the epigastric region, and sometimes on other parts of the body. And I saw a case myself, a few days ago, of a lady in a her 64th year, who had been laboring under what was supposed to be an attack of acute fever. On the 8th day of her illness, and just before she died, a large patch of erysipelatous inflammation was discovered under the left arm, and extending towards the left mammary gland. These patients generally had complained of thoracic or abdominal distress during their illness, and to this, without doubt, was due the cause of their death. There has been considerable reason to suppose, that in a number of cases, in which there were no external signs of erysipelas, the disease first commenced in some internal organ. Deep-seated, burning pain at the epigastrium, extending upwards and backwards towards the spinal axis, marked the commencement and progress of such cases, and pretty generally they went on to a fatal issue in a short time, attended with high fever and intolerable anguish. Completely developed on any portion of the body, the erysipelatous inflammation, after involving the dermoid tissue, extended to the contiguous muscles, to the eyes, ears, brain, bronchia, frontal, and maxillary sinuses; and sometimes to the viscera of the chest and abdomen; and, indeed, to every part in which there was any thing like a communication with the parts originally diseased. In the muscular system it was evinced by the tumefaction, soreness, and sloughing, which in some cases destroyed the muscles to a considerable extent around the parts diseased. When the inflammatory process ultimated in suppuration, the subcutaneous and inter-muscular cellular membrane seemed to be the parts first destroyed, after which the proper substance of the muscles becoming involved, very offensive sloughs were the consequence. Occasionally collections of pus formed between the skin and muscles after these parts had been detached by the ulcerative process. These depots of pus, if not opened early, usually enlarged, producing sinuses immediately under the integuments, or burrowing deep and wide among the muscles. Rarely a case of erysipelas of the scalp took place without the eyes being more or less implicated. Usually the inflammation was confined to the lids, by which the eyes were in most instances completely closed up. But instances did occur involving the ball of the eye; and in one case related to me by Dr. Camden, of Cedarville, one of the eyes was completely destroyed. A result so deplorable nevertheless was much less common than might have been expected, considering the frequency with which the eyes and parts auxillary were affected. To the internal ear, the affection was sometimes propagated, producing pain, imperfect hearing, tinnitus aurium, &c. The external ear was always very prominent among the parts involved in the inflammatory action; and it was generally through the meatus auditorius externus that the internal ear was invaded. I suppose it very probable that, in those cases where all the more urgent symptoms subsided, leaving a distress in the ear and head from which the patient could not be relieved, and which ultimately destroyed life, the cause of death was the extension of the disease to the brain. Evidently, however, the brain suffered in most cases from the great amount of irritation which occupied the face and scalp. In a case that terminated fatally a few days ago, the entire of the face and scalp was involved in a high degree of erysipelatous inflammation, which in the course of a short time gave rise to coma and muttering delirium.—These symptoms grew worse as the inflammation on the exterior began to give way, and continued to do so, until the patient expired. That the brain has sometimes been the primary seat of the malady, I have but little doubt; but the affection of this organ has more frequently appeared to be the result of an extension of the disease from other parts. Extension of the diseased action to the different sinuses was of occasional occurrence. It was evinced by deep-seated pain in the parts, accompanied in a short time with discharges from the nose and mouth of fœtid blood and pus. Complications of this character generally followed after a high grade of diseased action on the scalp, and were always of portending import. The frontal oftener than either the sphenoidal or maxillary sinuses suffered. This may in part be accounted for on the score of contiguity, the frontal being more immediately within the range of diseased actions than the other sinuses. Death, I have but little doubt, has often been the result of lesions kept up within these sinuses, after the urgent symptoms externally had disappeared, and the patient was confidently believed to be out of danger. I saw a case a short time since, complicated with inflammation of the sinuses. The urgent symptoms after five or six days, subsided, and convalescence was confidently expected. The truce, however, lasted for but a few hours, when typhoid symptoms supervened, and the patient died in a state of muttering delirium. Of the extension of the erysipelatous inflammation to the air-passages we had pretty plain evidence in a number of cases. Even where the disease was mild, unattended with any eruption, there were frequently present some very decided catarrhal symptoms. In my own person the catarrhal symptoms were very troublesome from the beginning. Cases of a more aggravated nature had a dry cough, burning sensations along the trachea, and at times difficulty of breathing. Some cases were complicated with pneumonitis, others with pleuritis.

Dr. Joshua Martin, of Xenia, had a case of erysipelas of the breast and arm. Very early visceral inflammation of the thorax supervened, and it was but a short time until the patient expired. Where the disease made its appearance in the chest, either primarily, or as the result of an extension, the pain attending it seemed to be deep-seated and burning, much worse, if possible, than can attend pneumonitis proceeding from ordinary causes. The supra-diaphragmatic portion of the alimentary canal, in all the varieties of the disease, seemed to be so naturally within the range of the diseased actions, that it appeared impossible for it to have escaped. With the exception of the soreness of the throat, which was usually present, we have seen no instance in which this or any other portion of the viscera were much diseased upon their mucous surfaces.

By what has been said upon the extension of erysipelatous inflammation from one portion of the body to another, or from one organ to another, we do not mean that anything like a metastasis takes place—that the disease was suddenly translated from one part to another. An example of this we have never witnessed; and we think its occurrence exceedingly rare. During the continuance of an inflammation upon the scalp and face, the brain became disordered gradually, and not apparently because the external inflammation had diminished, for this was usually not the case; but because the diseased action merely extended itself, increasing its dominions, without giving up any of its territory. The same is true of secondary disease of the thoracic viscera. The internal disease succeeded generally to an inflammation on the arm or exterior of the chest, and had no tendency to subvert the external. The only circumstance having any resemblance at all to a metastasis, was what once in a while took place in the throat. Sometimes we had the anginose distress very much mitigated, or wholly suspended, by the occurrence of erysipelas on the neck or face. This, however, was as easily accounted for on the principle of counter-irritation as by metastasis; and the former is doubtless the true explanation.

Having now sketched some of the more important features of the disease, as they were seen first on the skin, and from that organ extending to other parts of the body, I shall give the notes of a case or two, illustrative of the variety of which I have been speaking.

Case I.—Mrs. H. This lady was 50 years of age; her health previous to the attack of erysipelas had been impaired by a concealed intermittent, from which she had been but partially relieved. While in this condition she exposed herself in nursing her family who were sick with erysipelas. On the 6th of April, she was found laboring under the premonitory symptoms of erysipelas. Erysipelatous inflammation commenced first on one of the ears, and before twenty-four hours had elapsed, the entire of the face and scalp had become involved and the eyes closed up. The febrile excitement at first was slightly inflammatory, but not enough so to justify the use of the lancet. The patient was kept upon the use of hydragogue cathartics from the 6th to the 12th, with washes of acetate of lead and sulph. zinc to the local disease.

On the 12th, the phenomena were decidedly adynamic and the bowels also torpid. Wine, brandy, and barks were used to keep up the patient's strength, while injections were administered with a gum elastic tube, inserted high up in the bowels.

On the 16th, the eruption was found to be disappearing, but the general strength was more exhausted, and the bowels still remained unmoved.

On the 17th, the eruption had pretty much disappeared, but as this took place the frontal and maxillary sinuses, the throat and brain, seemed all to take on disease, giving rise to difficult deglutition, labored respiration, partial deafness, and muttering delirium. The case wore these symptoms until the 18th, when the patient expired. No post-mortem examination was made.

This case presents some of the various morbid phases of erysipelas when it is characterized by an eruption on the face or scalp. Its extension to the sinuses and brain giving rise to coma and delirium, and interrupting to such an extent the nervous influence, that keeps up the peristaltic action of the bowels, are consequences that are in perfect accordance with what *a priori* might have been expected. It is worthy of remark, also, that as the disease grew better on the exterior of the body, the internal trouble was increased.

Case II.—Mrs. B. between 50 and 60 years of age, quite corpulent, and of good constitution, had been engaged in nursing several persons laboring under the mild variety of the malady for several days previous to her own illness. On the the 21st of April, she complained of feeling unwell, and in the evening of that day she was taken with a chill, followed with fever. I saw her on the morning of the 22d, when she had thirst, a coated tongue, sore throat, pain in the head and back, and fever of the synochus grade. Immediately I gave her a large dose of cream of tartar and jalap, and ordered a gargle to the throat. The medicine operated well on the bowels, but the pulse, in the evening, remaining full and somewhat frequent, she was bled, until there was an approach of syncope. On the morning of the 23d, erysipelatous inflammation was developed on the end of the nose, and seemed inclined to extend towards the eyes. Corrosive sublimate in solution was applied to the part affected, and it was also surrounded with strips of blistering plaster about three-fourths of an inch wide, to see if the inflammation could not be arrested. Before the blisters had time to draw, the inflammation extended to the root of the nose, the eyelids, and to some extent, to the tunica conjunctiva. Very soon the nose, cheeks, and eyelids became œdematous, assuming the hue peculiar to the complaint. Finding the inflammation to progress irrespective of the means used, and the parts being exceedingly painful, I ordered a thin paste of cream and calcined magnesia. This was applied so as to paint over every part affected. On the application of this the patient expressed great relief. The relief proving temporary, I next had an ointment made of calomel and cream, but this was applied with no better success. On the 24th, finding that all local applications had been useless, and that the cerebral symptoms were becoming grave, attended with a sensation of tension, and weight upon the forehead, I concluded to try the effects of *incisions*. They were made tolerably deep upon the forehead and on each side of the face. The blood and serum flowed freely, and were promoted for some time by the applicatation of wet cloths. Immediately after this operation the patient expressed relief. The pain, tension, and swelling of the parts gave way, and against the 26th the appearances denoting convalescence were very decided. I shall proceed now to the consideration of what I think most proper to call

*The Lymphatic variety.*—The invasion and progress of this variety differ, in no material manner, from that which I have just described. Ushered in by the usual constitutional symptoms, such as languor, aching of the bones generally, pain in the head and back, rigors, and fever, it is not long, in the general way, before the lymphatic glands begin to show signs of passing into a pathological condition. Even in mild cases there was more or less predisposition in the lymphatic glands to become diseased. In my own case they were slightly enlarged on the anterior and lateral aspects of the neck. But in light attacks the swelling readily subsided under the use of discutients and sometimes spontaneously. Of a character, however, much more serious, were the disturbances in the lymphatic glands, when the influence of the remote cause was felt in the general system with more severity. In such the axillary and inguinal, as well as those about the neck, come in for a large share of the morbid action. Dr. Joshua Martin, and my partner, Dr. Winans, both politely furnished me with the results of their experience in this form of the malady. Dr. Martin witnessed four cases in which the morbid forces were principally spent upon the axillary glands, giving rise to swelling, induration, and suppuration, as well of the glands, as of the parts contiguous, including the skin, cellular tissue, and muscles. Most of the time the glands were exceedingly painful, and as the process of suppuration came on and progressed very sluggishly, the system was kept in a feeble, irritable state for weeks, the physician being unable to do any thing, except palliate suffering until the disease had run its course, and terminated in suppuration. Three cases, all of which occurred in the same family, were observed by Dr. Winans; in all of which the same trouble in the axilla obtained. The enlargement and induration continued, as in the cases to which we above alluded, for some time before suppuration took place; once in a while it was the case, that there was a kind of transient erythema of the shoulder and breast, associated with cases such as we have been describing; and some instances did occur, where the erythema became permanent, and in the latter stage passed into phlegmonous erysipelas, ulceration, and sloughing of the muscles and other parts contiguous. A combination nevertheless, of the erysipelatous eruption and severe disease of the lymphatic glands, was not, we think, of common occurrence. Usually when the disease was found betraying a preference for the lymphatic glands, the skin was exempt. Less frequent also were complications, when the lymphatic glands were the principal seat of the malady. The suffering endured, however, was greater. Afflicted for a long time with subacute and chronic alterations in the glands, patients became very much emaciated and suffered much more than was usual for others with an acute form of the malady, and more or less eruption on the skin. Completely seated upon the lymphatic glands, I saw or heard of no case, in which, by the use of any means, the enlargements were removed; suppuration was the inevitable consequence; and this as before remarked, took place in a very gradual manner. I will now transcribe from my note-book a case which may be regarded as a pretty fair specimen of what the disease is when it principally affects the lymphatic system.

The case occurred to a child, aged 11 months. It was taken unwell on the 29th of September last, with restlessness, thirst, and fever; and in the evening of that day it took a dose of oleum ricini, which produced two or three discharges from the bowels.

Oct. 2d. To-day I find present the following phenomena: considerable fever of the inflammatory kind; erythema upon the face and neck, and to some extent on the body; the lymphatic glands under the right arm swollen and painful, involving the pectoralis major; tongue furred; fauces red; and the lips raw. Prescription—an emetic of ipecacuanha followed by pulvis Doveri.

Oct. 3d. The fever rather typhoid; pulse small and frequent; erythema disappearing on the face and neck, but on the body and extremities there is an eruption consisting of very minute vesicles filled with a transparent fluid; axillary glands enlarging. An emetic, hydrarg. cum creta, and an anodyne cataplasm to the axilla.

Oct. 4th. No change, except that the anomalous appearance on the skin is more general. Bitartrate of potassa sufficent to keep the bowels soluble.

5th. The eruption is disappearing; the glands in the axilla remain swollen and painful; pulse soft and small; throat better. Cinchona and rhubarb.

6th and 7th. The patient continues in the same state.

8th. The tumor in the axilla is discharging a bloody matter mixed with whey-like fluid. From this date the patient was kept on the use of aperients and strengthening medicines. The discharges from the axilla continued for near two months, during most of which time the patient was feverish and irritable; and as the ulcer seemed to be a kind of outlet, established by the powers of nature to drain the disease from the general system, nothing but emollient cataplasms were applied to it, until convalescence was established.

*A case in which the tongue was the principal seat of the malady.*

In some regions, I have reason to believe, much more than in my own, the disease has betrayed a preference for the tongue. This organ, I have noticed, in many cases where it was not prominently diseased, was affected with more or less pain about its root, at times shooting through the substance of the organ, giving rise to stinging sensations, and at other times extending upwards and backwards along the course of the eustachian tubes. But neither the frequency nor the severity with which the organ has been affected, is sufficient to justify those revolting names that have been so rife in every section of the country in regard to "*black-tongue.*" Having witnessed a case that perhaps displayed the phenomena ordinarily present when the tongue shares prominently in the diseased actions, it will now be submitted.

W. G. Y. The subject of this case was a man of strong constitution, plethoric habit, and 46 years of age. He had been waiting upon different members of his family, afflicted with erysipelas of the mild and malignant varieties, for two weeks previous to his own illness. During this time he became once or twice indisposed, but by taking cathartics his health was restored.

On the 4th of May the following symptoms were present: aching of the bones generally; pain in the head and back; sore throat; loss of appetite; tongue slightly coated; chills, and flashes of heat. A dose of calomel worked off with senna.

In the evening, twelve hours after the first examination—the pulse is now full and frequent; countenance bloated; breath hot and fœtid, skin bathed in a copious clammy perspiration. Bled to 24 ounces, and gave a full dose of cream of tartar and jalap.

5th and 6th. Symptoms somewhat moderated.

7th. The respiration is hurried, at times being difficult, and a deep-seated burning pain is felt in the lower and posterior part of the chest. Bled *ad deliquium*, and a full dose of pulvis Doveri. Blood neither in this nor the former bleeding buffed.

8th. To-day, for the first, he is complaining of his tongue. It presents a healthy appearance, except that it is slightly coated, and the papillæ on one side of it are slightly enlarged. A saline aperient, and mouth wash.

9th. The pulse is now smaller than it has been and rather more frequent; the tongue is painful and considerably enlarged, though it has not altered its color, and the secretions from it are abundant. The patient is unable to lie down, owing to the circumstance that the tongue falls back on the fauces and impedes his breathing. Incisions made into the upper and lower surfaces of the tongue.

10th. The tongue continues to enlarge.

11th. It now occupies almost the entire cavity of the mouth, and is protruded for some distance between the teeth, from which latter circumstance the jaws are forced apart, and the patient sits all the time with his mouth open unable to articulate; its lower surface, where the scarifications were made, is covered with a thick dense layer of false membrane; its color is redder than on the 9th, though the secretions from its surface, and from the inside of the mouth, and salivary glands, are very copious; the pulse is tolerably full and resists pressure. Bleeding to about 20 ounces, and an emetic of ipecacuanha and common salt, by advice of Dr. M. Chambers. Blood quite much coated.

12th. To-day there is a slight remission of all the more urgent symptoms, and the patient thinks he feels better. An emetic of ipecacuanha and common salt, together with a Seidlitz powder.

13th. The swelling of the tongue is beginning to subside; the false membrane is getting loose and readily peels off; the fever is also less than formerly. A Seidlitz powder.

On the 14th, the patient was still found improving, and under the use of a gargle of common salt and aperients his health was restored.

Various local remedies were applied to the tongue in this case during the time it was so much enlarged, such as solutions of nit. silver, sulph. zinc, and alum, Velpeau's favorite remedy in sore throat; but nothing made anything like a curative impression. Common salt exercised as favorable an influence in cleansing the tongue and fauces as anything that was tried. And to allay the tormenting irritability with which the tongue at times was affected, mucilaginous preparations of slippery elm, and gum arabic were used.

Of a character altogether the most interesting, because attended with the greatest mortality, are those cases showing

THE CONNEXIONS OF ERYSIPELAS WITH THE PUERPERAL STATE.

Having heard that the disease had prevailed in Warren and Montgomery counties, with very considerable malignity, and that women in the puerperal state had suffered more than any other class of persons, I concluded to make a visit to those regions, for the purpose of obtaining information. At the time that I arrived there, which was in February last, the disease was not prevailing. From Drs. Adams and Strong, of Centreville, and Drs. Vantuyl, Steel, and Jewett, of Dayton, I however learned some of the more important facts connected with its prevalence in their vicinities. These facts, as they were verbally detailed to me, in an interview with the above named physicians, will now be given.

Dr. Strong had one case of erysipelas in the puerperal state. He had been attending to a case of erysipelas in which there were suppuration and great prostration of the general system. During his attendance on this case he was called to a lady about to be confined in child-bed. He attended to the accouchement, and in about 12 hours afterwards the lady began to complain of pain in the abdomen. The abdomen became very much distended, the pulse frequent, with high fever, and in seventy-two hours the patient died. After this Dr. Strong took erysipelas himself. and saw no other case of the above description.

During the prevalence of the epidemic Dr. Adams, on account of the illness of Dr. Strong, had a large share of the practice at Centreville and in the vicinity; and as a consequence, he witnessed some important phenomena in connexion with the puerperal state. Here is an abridged account of the puerperal cases which fell under his observation.

Case I. The doctor went from an erysipelatous patient to a lady taken in labor, aged 25. The labor was natural, and the child was delivered without any difficulty. In 24 hours after delivery, a chill announced the approach of disease; and in a short time high fever followed, the abdomen became swollen and tender, and the patient died on the fourth day.

Case II. Went to this case after visiting an erysipelatous patient. The constitution of this lady was rather feeble. She, however, had an easy labor; but in twenty-four hours after her accouchement she was taken with a chill, high fever, great distention and tenderness of the abdomen followed, and she died about the fourth day.

Case III. Saw this case immediately after visiting an erysipelatous patient. This lady was aged 40, of good constitution, and had an easy labor. In 28 hours afterwards, she took a chill, the pulse became irregular and small, abdomen distended and tender, and she died in 24 hours from the time she was taken.

Case IV. Dr. Adams was called to this case from a patient sick with erysipelas. The lady was 30 years of age, of good constitution, and had an easy and speedy labor. She was seized with a chill in 32 hours after delivery; her pulse became very full and frequent, abdomen painful and much distended, and she died on the fourth day.

These cases occurred in the practice of Dr. Adams in succession, one after another, at the time at which erysipelas prevailed in the neighborhood. When the epidemic subsided puerperal disease also subsided.

Dr. H. Vantuyl, of Dayton, favored me with the following facts in regard to erysipelas as it was manifested in his practice among females in the puerperal state.

Case I. This lady was aged 28, and of good constitution. She was taken in labor with her first child and had an easy delivery. In about 35 hours after delivery, she was seized with a burning sensation just below the ensiform cartilage; the pulse was quick, and there was also some fever; there was no trouble apparently in the abdomen. She died in 24

hours from the time of attack. Dr. Vantuyl thought the cause of her death was inflammation of the diaphragm.

Case II. This female had a good constitution, and was healthy up to the time of parturition; she was the mother of three children. Thirty-eight hours after delivery, she was taken with a burning sensation near the ensiform cartilage which extended upwards and backwards towards the spine; the pulse was 120; no pain or distention of the abdomen. Died in 48 hours from the time of attack.

Case III. This occurred in a female aged 30 years, and of good constituton. Her labor was easy and not at all complicated. Twelve hours after her accouchement she commenced complaining of pain in the region of the mediastinum; the pulse was small but very frequent, 130 to the minute; the excitement appeared congestive; and she died in 12 hours, apparently from suffocation.

Case IV. The health of this patient had been good previous to her accouchement. There was no distress of any kind in the region of the uterus or upon the abdomen, except in the epigastric region, which she said was the seat of severe pain; the pulse 130, and the excitement congestive. She died in a very short time. After death an erythematous blush made its appearance over the part where she had complained of feeling so much pain.

Case V. The constitution of this patient was good, and her health had remained unimpaired until she had arrived at about the expiration of the sixth month of utero-gestation, when she was seized with a burning sensation in the epigastrium. Shortly after this labor pains commenced, and an abortion succeeded. Twenty-four hours after the abortion she died. After death an erysipelatous redness made its appearance on the epigastrium and extended around on one side towards the spine.

Case VI. This patient had good physical powers and was in the enjoyment of her ordinary health at the period of parturition. At the time she was delivered she complained of pain on the *labia externa*. In a short time erysipelatious inflammation was developed on this part, and extended over the vulva and perineum, and to the insides of the thighs. The fever was high and the erysipelatous inflammation very acute. There was no tenderness, pain, or swelling of the abdomen. Forty-eight hours after the patient was taken she died. In this case there was considerable sloughing from the perineum and insides of the thighs.

Case VII. This patient was of nervous temperament, and the mother of four children. Her accouchement had been managed by a midwife. In about 48 hours after delivery, she was taken ill, and when Dr. Vantuyl saw her he ascertained that she had first complained of pain in the hypogastric region. This was followed by the development of erysipelatous inflammation on the abdomen and thighs; and it was but a short time after this until she died. The fever in this case was congestive, the excitement being confined to deep-seated organs of the body.

During the prevalence of the epidemic, Dr. Vantuyl attended to about 40 cases of labor, all of which escaped the malady except those above given.

Dr. H. Jewett gave me the following facts in relation to the puerperal cases which occurred in his practice.

Case I. This lady was of tolerable constitution, aged 35. At about the 7th month of gestation, she took a sore throat, which was followed by considerable fever, and an abortion. Very soon erysipelas made its appearance on the breast and superior extremities, attended with a very œdematous condition of the parts. Delirium supervened, and the patient died on the seventh day.

Case II. This lady, aged 30 years, had previously enjoyed good health. The accouchement had taken place before the doctor saw her. From the friends, however, he learned that the labor had been uncomplicated. After delivery she complained of sore throat, and a sense of pain on one of the arms. Immediately after this the abdomen became painful, and very much distended, and she died in twenty-four hours after delivery.

Case III. Was a female who was healthy up to the time of parturition. Her labor was uncomplicated; but in 48 hours after delivery the abdomen commenced swelling, the pulse grew very frequent and rather small, and in 48 hours more she expired.

Dr. Jno. Steel informed me that he witnessed six or eight fatal puerperal cases complicated with erysipelas. Symptoms of erysipelas were manifested in all of them in from 12 to 24 hours after delivery. All of them had the pulse and febrile orgasm peculiar to puerperal fever. Seldom was there any pain in these cases that could be strictly referred to the uterus. There nevertheless were great pain, tenderness, and distention of the abdomen in all the cases Dr. Steel witnessed.

January, February, and March, 1844, was the period selected for the epidemic to make its appearance in Dayton, Centreville, and their vicinities. Pretty much all the cases of parturition attended to by Dr. Adams, of Centreville, during the time of the epidemic, were seized with the complaint. Dr. Steel thought the proportion of puerperal cases, when compared to the whole number of accouchements to which he attended, was about one to five. In Dr. Jewett's practice it was about one in twelve. In Dr. Vantuyl's one in ten.

As it regards the age of females most predisposed, nothing definite was observed. All ages suffered pretty much alike. Women, who had just entered the period of child-bearing, were as liable as those who had advanced pretty well towards the close. Of the habit most predisposed but little could be told. Dr. Steel thought the scrofulous and phthisical most liable. Other physicians, however, could see no difference; and the cases detailed above, show, with but one or two exceptions, that the healthy and those of good constitution were the principal sufferers.

Various as were the periods of attack from the time of delivery, they nevertheless in the majority of cases were short

In from 12 to 48 hours the symptoms were usually developed. Brief to equally the same extent, was the duration of the malady. In several instances it ran its course in twelve hours. A few cases attended with extensive sloughing lasted for five, six, or seven days. The majority, however, died in 48 hours from the time they were taken.

Deeply to be deplored has been the mortality incident to erysipelas in the puerperal state. Of all the cases which occurred at or near Centreville, or in Dayton or vicinity, not one recovered. Such also has been pretty much the result of similar cases wherever the disease has prevailed.

As a matter of serious inquiry, the question might be here started, whether the disease we have been considering is a mere variety of puerperal fever, or whether it is identical with epidemic erysipelas, deriving its malignity from the peculiar state of the system found at the period of parturition? From what is known respecting the etiology and modifications of puerperal fever, it must be admitted that it has been known to originate from a variety of causes, becoming at times epidemic, and assuming various phases of morbid action. Still I do not regard the malady under consideration, as being identical with any variety of puerperal fever, but as being genuine epidemic erysipelas, supervening upon the puerperal state, and giving rise to some phenomena more or less analogous to puerperal fever. In confirmation of this position it might be submitted:

1. That the disease prevailed at the same time that erysipelas was prevailing, and subsided on the disappearance of that malady.

2. That the premonitory symptoms were like those of erysipelas, consisting of a chill followed by more or less fever and sore throat.

3. That in several instances, erysipelas made its appearance on the skin during the progress of the malady, as was the case with several families witnessed by Dr. Vantuyl and others.

4. Instances occurred in which erysipelas was contracted from puerperal females by their nurses.

5. That the children delivered in almost every instance, were taken, shortly after birth, with high fever, erysipelas of the scalp and face, and died.

Facts of this character must be regarded as going to settle in a tolerably decided manner, the position, that the fatal disease which occurred to the puerperal females, to whom I have alluded, is identical with the epidemic erysipelas which prevailed at the time in the neighborhood. Any body, indeed, witnessing the epidemic, would have failed in coming to any other conclusion. The evidence in most cases was not of the probable kind; it seemed to be entirely positive. I should not have dwelt on this subject, further than to have given it merely a passing notice, had it not been that European, and to some extent, American writers, have spoken upon it in terms barely of suspicion. They mention the prevalence of erysipelas during the time that fatal puerperal disease was committing its ravages, but refer, so far as I have learned, to no facts, for the purpose of establishing an identity.

Into a consideration of the contagious nature of the malady, and of the question whether the virus was capable of being carried by the physician from one parturient female to another, although inquiries, exceedingly interesting, I do not expect to enter, further than merely to state a few facts. Of the circumstances going rather against such an hypothesis we may state,—

1. That there were a great many females, who were attended to by physicians practising in epidemic erysipelas, that did not contract the malady at all. It should be recollected here, however, that all, or nearly so, of the cases managed by Dr. Adams, of Centreville, terminated fatally. But such was not the case with the physicians in Dayton. Of those delivered by Dr. Vantuyl, about one in ten took the malady; of those by Dr. Jewett, one in twelve; and those by Dr. Steel, one in every five. My partner, Dr. Winans, and myself have attended to some cases of parturition while we were practising in epidemic erysipelas, but as yet no puerperal disease has been developed.

2. The proportion of parturient females that contracted erysipelas was not much above that of any other class of persons. In localities where the disease prevailed all classes were more or less affected with some form or variety of the malady; and it is problematical whether any of them enjoyed an exemption greater than females in the puerperal state, only about one in eight, taking the whole district into consideration, having suffered. Now I believe this is a proportion not far from correctness when applied to other classes during the time the disease was epidemic.

3. Several females contracted the disease spontaneously anterior to the period of parturition, and in a case or two it produced abortion.

Favorable to the supposition that the disease was contagious, and that the poison was carried by physicians from severe cases of erysipelas to parturient females, are the following circumstances:

1. All the females afflicted, were taken ill and died during the time that erysipelas was at its height.

2. Puerperal disease commenced shortly after the appearance of epidemic erysipelas, and was synchronous in its prevalence with that disorder.

3. The four cases, which occurred in Dr. Adams's practice followed in succession, and that, too, while he was visiting hardly anything else in the neighborhood but erysipelatous disease. It is a fact also that in Dayton, when the physicians had the greatest run of business in epidemic erysipelas, nearly all the cases of puerperal disease were developed.

4. Females delivered by the midwives of the district, although not entirely, were much more frequently exempt, than those delivered by physicians having a number of erysipelatous patients.

5 Physicians in the districts adjoining, who had no erysipelatous patients, saw nothing of the disease among puerperal women.

6. Popular opinion, though not generally entitled to much confidence, is nevertheless, at times, founded upon circumstances, that should be scrutinized. In the matter before me, the females of the district, who had not arrived at the full period of gestation, came to the conclusion that there was danger in permitting physicians, who had been practising extensively in erysipelas to deliver them. They were of the opinion, that physicians carried the disease from one patient to another in their clothes, and hence, after they had ascertained that a physician had attended to some fatal cases of puerperal disease, or been practising in other forms of erysiyelas, his services were pretty generaly declined. I mention this circumstance as a mere matter of history, rather than as being entitled to much consideration. Popular opinion is very good authority when it is right. But it is so seldom founded upon circumstances entitled to regard, that in medical matters it can scarcely ever be recognized as an element of evidence.

It may not be out of place to submit here some evidence touching several of the points under consideration, from various regions of my own country, and some also from foreign countries.

On consulting the Quarterly Summary of the Transactions of the College of Physicians of Philadelphia, May, June, and July, 1842, as published in the American Journal of the Medical Sciences, it will be found that Dr. Condie called the attention of the College to the prevalence at that time of "puerperal fever of a peculiarly insidious and malignant character. The disease was mostly confined to the southern sections of the city of Philadelphia, and to the lying-in wards of the Blockley Hospital. It was a fact worthy of notice that in the neighborhoods and even houses in which cases of puerperal fever occurred, erysipelas has prevailed to a greater or less extent. Erysipelas has indeed been far more prevalent throughout the whole of the districts south of the city during the past winter and spring, than it has been known to be for the last twenty-six years.

As it regards the contagious nature of this affection, Dr. Condie remarks as follows:

"An important query presents itself in reference to the peculiar form of puerperal fever now prevalent. Is it namely capable of being propagated by contagion; and is a physician who has been in attendance upon a case of the disease warranted in continuing, without interruption, his practice as an obstetrician?"

Dr. Condie was not a believer in the contagious character of many of those affections generally supposed to be propagated in this manner, but he nevertheless became convinced by the facts that had fallen under his notice, that the puerperal fever which had prevailed was capable of being propagated by contagion. "How otherwise can be explained," he remarked, "the very curious circumstance of the disease in one district being exclusively confined to the practice of a single physician, a Fellow of this College, extensively engaged in obstetrical practice, while no instance of the disease has occurred in the patients under the care of any other accoucheur practising within the same district. Scarcely a female that has been delivered by this gentleman for weeks past has escaped an attack.

Dr. Huston, obstetrician to the Philadelphia Hospital, and Fellow of the College, spoke of puerperal fever as being epidemic in the institution of which he had the charge, and remarked that he had noticed at the time quite a tendency to erysipelas. Dr. Houston gave no opinion relative to the contagious nature of the complaint.

Present at the same meeting of the College of Physicians, was Dr. Warrington. He saw several cases of the disease of which the members had spoken; and two instances in which the nurses of the lying-in females had contracted erysipelas, one upon the scalp and face, the other upon the leg; the former died, the latter recovered. This I may remark, by the way, was pretty strong evidence, as well of the identity of the diseases, as on the question of contagion.

Dr. West stated some facts related to him by Dr. Jackson, of Northumberland, but at that time of Philadelphia. It seems that while practising in Northumberland county, and at a time when erysipelas was prevalent, Dr. Jackson delivered several females in rapid succession, all of whom were attacked with puerperal fever. "Women," Dr. Jackson observed, "who have expected me to attend to them now becoming alarmed, removed out of my reach, and others sent for a physician residing several miles distant. These women as well as those attended by midwives did well; nor did I hear of any deaths in child-bed within a radius of 50 miles, except two, and these I ascertained to be caused by other diseases. I now began to be seriously alarmed on the score of contagion. Although I had used some personal precautions before, I now feared they had not been sufficient."

Dr. Stewardson observed, that in 1830, while he was resident physician of the Pennsylvania Hospital, and had erysipelas prevailing in the surgical ward of that institution, a very malignant puerperal fever made its appearance in the lying-in ward; and that all the patients died. Nothing, he said, arrested the progress of the malady, but the entire evacuation of the wards, and the closing of them against further admissions.

In his Retrospect, No. ix., 1844, Braithwaite published a paper from Robert Storrs, Esq., of Doncaster, in which is contained a considerable amount of evidence on the subject to which we have alluded. Mr. Storrs enumerates a number of instances, in which a singularly malignant form of puerperal fever was produced in lying-in females by having physicians to attend them, who were at the same time visiting cases of gangrenous erysipelas. It seems also that Mr. Storrs had written to a number of gentlemen for information. Now on this correspondence he remarks as follows:

"I would here briefly draw the attention of the reader to the remarkable and striking fact of Mr. Reedal having five fatal cases of this horrid malady in labors which he attended, so immediately after dressing a case of malignant erysipelatous disease, and on his leaving off attendance on this case having no more of it, and that neither his pupil, nor any other medical gentlemen in Sheffield having any other instance of it among their labor cases; also that Mr. Sleight had two cases of it while in attendance on a case of erysipelas; that Mr. Hardey's cases also arose while he was in attendance on a case of sloughing abscesses and of erysipelas; and again, that three surgeons were simultaneously the means of spreading puerperal fever from a post-mortem examination of a case of gangrenous erysipelas, a combination of evidence I think sufficient to convince the most sceptical that this disease produces a subtle animal poison, which is instrumental in propagating, when puerperal women are subjected to its influence, whose predisposition favors it, a disease in about thirty-six or forty-eight hours afterwards of the most inflammatory, prostrating, and violent character—a disease which stamps death on the features and in the symptoms immediately on its occurrence."

After announcing the above facts Mr. Storrs adds by way of advice the following:

"As it is well to be always guarded against such misfortunes, I think it desirable for midwifery practitioners to avoid attending labors in the same clothes in which they attend their ordinary patients, especially the coat, as this garment must be the one most likely to be the means of conveying fomites; and at any suspicious period, when typhus or erysipelas is prevailing, to carry out the same carefulness even in the after-attendance on labor cases."

From the character of this evidence, and much more equally strong, found in the records of the profession, the question of contagion assumes a very grave form. If not entirely established, it may at least be regarded as verging very fast beyond the point of cavil. True it is, that all the cases to which we have alluded may, by some, be accounted for on the score of coincidence. But would this be a safe conclusion? I opine not. The facts against it are too strong and numerous, and, in my estimation, sufficient to make a decided preponderance—sufficient to induce practitioners of medicine, when they have a sloughing case of erysipelas to be ware, not only in regard to their visits to parturient females, but also as it regards their intercourse with other patients.

Incidentally in a previous part of this essay, I mentioned the fact that most of the children of lying-in females contracted the disease and died. Some of these, it would seem, were born with symptoms of erysipelas, while others showed no evidence of disease for some time after birth. As far as I have learned, the disease first manifested itself upon the scalp, extending from this to contiguous parts, including in most instances the brain. The course was generally very rapid, a few days, at most, being sufficient for the malady to do its fatal work.

### CAUSES REMOTE AND PROXIMATE.

As it regards the manner in which the contagion was originated and propagated, our ideas must, in the present state of medical knowledge, remain more or less vague. Something like what Hippocrates would call an *erysipelatous constitution of the atmosphere*, has no doubt obtained in most of the States and Territories in the Mississippi Valley. From a great many places in this extensive region, we have received information that the malady has prevailed, or was prevailing, attended with various grades of mortality. It is true that there are many localities in which it has not yet made its appearance. But from the territory already embraced, exemption from its influence cannot reasonably be expected at any place west of the Alleghany mountains. It commenced almost simultaneously at several points, and has gradually been extending itself, until at the present time it is epidemic in more places than it has been at any time previous. How otherwise can its presence in these various localities be explained, than by adopting the idea of Hippocrates and Sydenham, viz: that it depends on an epidemic constitution of the atmosphere? By this, however, we mean nothing more than that the disease has not been propagated; but that like cholera and other epidemics, it seems to owe its introduction to a certain *constituo æris*, about which we know nothing, further than what may be learned from its effects. The malady was first introduced into our region from a known source of contagion. But all the cases contracted from this source recovered; and we saw nothing more of it for nine or ten months, when it again made its appearance simultaneously in several different neighborhoods at about the same time.

Generally when it made its appearance in a family all the members thereof became sooner or later affected. Those engaged as nurses seemed most liable. Those who spent much of their time in the sick chamber were also very apt to be seized with the complaint. Visitors, though seldom, yet, once in a while were affected, showing that mere exposure to the source of contagion, under some circumstances, was sufficient to bring on the malady. The time of attack, after being exposed to the virus, varied from three or four days to two weeks, and I saw the case of a young lady who did not contract it until three weeks had elapsed. So far as the origin of the virus, by which the disease is spread to the different members of a family, is concerned, it may be remarked in general terms, that there are a great many diseases, which on becoming malignant, are capable of elaborating a peculiar kind of virus, that will produce the same disease in others exposed to its influence. This has been found to be the case relative to many diseases classed under the head of pyrexia, as well as to some of those classed under the name phelgmasia. How, or in what manner, the contagious substance is generated, is one of those questions which bid defiance to human research. After the poison is once generated Liebig's mode of accounting for its propagation is the most philosophical of any that has fallen under my observation. He adopts in explanation the law of dynamics, that "a molecule set in motion is capable of imparting its own kind of motion to any other molecule with which it may be in contact." Hence he concludes that after contagion is once generated and comes in contact, either in the gaseous, liquid, or solid form with any part of the organism susceptible to its ininfluence, as for example, the mucous membrane, or an abraded portion of the skin, it is capable of imparting its own kind of motion or diseased action to the parts, in a manner similar to the transforming process of eremacausis or of fermentation. This explanation may look rather too mechanical; but when it is recollected that it is in this way, that we at pleasure produce the vaccine disease, and also variola; and that a piece of mortified or diseased flesh will, in many instances, occasion disease and death if applied to a flesh-wound, such objections will have for the present to be waived, at any rate, until a more philosophical explanation of the matter is proposed.

*Proximate Cause.*—In the English language, there is no work, not excepting the learned one of Prof. Gross, that to any extent discusses the pathology of *sporadic* erysipelas; and *epidemic* erysipelas owing to the fact that it has been of rather rare occurrence, has hardly received a passing notice. Hence, in his work on *Diagnosis*, Marshall Hall says, "I need scarcely repeat what I have already stated several times, that the morbid anatomy requires to be investigated anew in reference to each of the eruptive fevers; this remark applies to erysipelas, equally with scarlatina, variola, &c." All, therefore, that is known of epidemic erysipelas, as it is seen in its different varieties, is the result of very partial dissections and clinical observation. And as I have already, when speaking of the different varieties of the disease, anticipated much of what usually falls under the head of proximate cause, it will in this place be unnecessary to do anything more than make a brief recapitulation.

1. The onset of the complaint was pretty much alike in all the different varieties, consisting of lassitude, aching of the bones generally, chills, followed sooner or later by fever.

2. As a general remark, the pyrexial excitement was at first of the synochus grade, very amenable to the lancet; and when the disease became complicated with local affections, protracting its course, the fever became typhoid.

3. Usually the blood drawn had a rich firm appearance, the very opposite to that found in typhus and other adynamic diseases. The blood, however, was not at the commencement of the disease buffed. I drew blood from a patient laboring under a high grade of arterial excitement, that for the first two bleedings presented no size whatever upon its surface. After these bleedings the patient's tongue became inflamed and hypertrophied, and on the third bleeding the buffy coat was quite thick. The bleeding of this case confirmed, in a very striking manner, the position of Andral, that in diseases strictly febrile, the blood never presents the buffy coat; but, that no sooner than inflammation is added, than the the fibrin gets into excess, and the buffy coat is developed. During the time of our first and second bleedings of the case, to which I have referred, I have no evidence of the existence of anything but fever; but at the third bleedmg the tongue was very much inflamed. Concerning the clot and serum, Andral's remarks were also found to be true. When the patient had fever alone, the clot and serum were imperfectly separated, and the latter existed in a small proportion. No sooner, however, was the local disease developed, than the clot became smaller and the serum existed in much greater proportion.

4. So various were the anatomical conditions produced by this affection in different individuals, and even in the same individual, that we fear it will be found very difficult to arrive at any positive conclusions concerning the pathology proper. Altogether of the most constant occurrence in every variety ol the malady was the disturbance found in the lymphatic glands. I witnessed but few cases in which it was not more or less conspicuous. In what is termed the mild variety, the trouble consisted in nothing more than a partial hypertrophy and induration of those situated on the front and lateral parts of the neck. With such I had but little trouble; the tumefaction usually subsided on the use of remedies addressed to the general system. In cases where the remote cause operated with considerable severity, it affected the glands in a manner much more grave. In such, the malady seemed to spend its principal force almost exclusively on the lymphatic glands, leaving the skin and other portions of the organism in which the disease is sometimes seen, unaffected. Upon the glands of the axilla, the disease was most frequently seated, though I learned that in some districts it was oftener felt upon those of the inguinal region. With the peculiar character of the alterations which took place in these diseased glands, we are unacquainted, except with what could be learned from an examination during life. Very soon after the system was invaded by the morbid forces, complaints were made of soreness and pain in the parts. At first the pain was severe, denoting the presence of an acute inflammation, and although some degree of mitigation was produced by emollient poultices, patients nevertheless complained very much throughout the whole course of the disease. The hypertrophy and induration were always very considerable, and in some instances great. These pathological conditions, after lasting an indefinite length of time, were succeeded by suppuration, sloughing, and discharging from the glands of ill-concocted pus. It was very obvious that the complaint, as it manifested itself in these glandular swellings, had a certain cycle of changes through which it would run, and from which, by no remedies that we are acquainted with, could it be removed. When the disease of the general system was slight, or readily amenable to treatment, we had no trouble with the glands; but, on the contrary, when the morbid forces were felt in the system with great severity, there was a corresponding violence in the local affections.

5. From the fact that the term erysipelas is associated in our elementary works with diseases peculiar to the skin, it might hence be inferred that this organ, in the disease we are now considering, is invariably the seat of all the more important pathological elements. Such, however, is not the case. In the simple variety there was much less functional derangement than is usually witnessed in continued fever. Very frequently patients perspired spontaneously throughout the whole course of the malady, and when this was not the case, the mildest diaphoretics were capable of exciting the perspiratory exhalents into vigorous action. Nor was it usual for this variety to present any structural alterations of the skin. To nearly the same extent is what we have said of the mild form, true of all the cases where the glandular system was prominently implicated. Now the proportion of cases, which, in my experience, fell under these two forms, when compared to the whole number affected, was as nine or ten to one; and in some districts it was much greater than this. When there were any lesions of the skin they varied very much in different cases. In some the alteration consisted of a mere erythematous blush, indicating that Good's classification is correct. In other instances there was no erythema at all; but the skin presented a pale appearance, with very minute elevations of the cuticle, containing a transparent glistening fluid. The character of the eruption, in these cases, indicated, to some extent, the classification of Willan, who includes the disease under the order bullæ, to be correct, were it not for the fact that bullate eruptions are pretty generally supposed to be associated with an inflamed skin, which, in the instances to which we have alluded, as has already been remarked, was not the case, the skin being abnormally pale. In another class of patients, the skin, on the third or fourth day, became violently inflamed with a tense, shining appearance. Upon this inflammation bullæ, from the size of a pin's head to that of a shilling, or larger, were more or less numerously developed, similar in appearance to those produced by a blister of cantharides. Following this, there were great tumefaction and pain, attended with the effusion of serum beneath the skin, and between its different laminæ. The effused serum at times assumed a peculiarly ichorous character, which, on pervading the dermoid tissue and contiguous parts, gave rise to what is usually called *erysipelas phlegmonodes.* These facts in relation to the lesions which have been observed in the skin, warrant us in concluding:

*First*, that in nine-tenths of the cases of epidemic erysipelas, the skin is not structurally affected; and secondly, that when it is diseased, there is nothing like uniformity of appearance in the character of the eruption, some being affected with a mere erythema that appears and disappears several times during the twenty-four hours, others with bullæ, exceedingly small, seated upon a pale skin, and others presenting the true erysipelatous eruption.

6. To the stomato-pharyngean mucous membrane there pretty uniformly occured more or less disturbance. Early in making its appearance, it was also of constant attendance. As yet I have seen no case in which it was not present in some stage or other of the malady. And hence if there be one lesion which more than another is entitled to the character of a pathognomonic symptom, it is found in the throat. True, in many cases where the symptoms were violent from the beginning, it was the case that the anginose distress was so completely masked by those of an urgent character that it was liable to be overlooked. A close critical examination, however, we believe, would have revealed it in every case. The nature of the affection in the mouth and throat varied in different individuals. In nearly all it consisted at first of a dryness and painful state of the lining membrane of the fauces, mostly confined to where the membrane is reflected over the tonsils, velum pendulum palati, and uvula; but occasionally extending further back into the pharynx, or forwards and upwards into the roof of the mouth or posterior nares. Examination disclosed the parts to be of a bright red color, denoting a pretty high grade of arterial action; but owing to the fact that the affection of the fauces, in almost all instances, was of short duration, abrasions of the mucous surfaces, or secretions of false membrane were very rare. When the integrity of the parts was assailed, there was much less tendency to run into gangrene or protracted ulceration than is usually witnessed in angina maligna. The local arterial action was more of the character of an active hyperemia, and preserved this trait pretty much throughout the whole course of the disease.

7. To the *tongue*, most practitioners, on the first appearance of the epidemic, looked with considerable solicitude. This was in consequence of the disease, in some regions, having been provincially named "*black tongue;*" and hence it was expected that the tongue would be the most constant seat of the malady. Such expectations were not realized. Commonly the tongue was foul, but not more so than it is found to be in other diseases of the same grade of violence. It was also in many cases the seat of painful sensations about its root when the fauces were much inflamed. No instance was seen in the Miami valley, as far as I have heard, in which it was affected with anything like an erysipelatous eruption. The case which has been detailed in a previous part of this paper, and which is the only one which occurred in this district, where the tongue was the prominent seat of the disorder, was not affected in any of its stages with an eruption. The organ was pale at the beginning, and even after it commenced swelling it underwent but little change, so far as its color was concerned, for some time. A tingling sensation was felt near the apex for several days before the enlargement commenced. During the time it was swelling, nothing externally was observed to which such a grave lesion could, with any propriety, be ascribed. The morbid forces seemed to me to commence upon the internal substance of the organ, and from this pain extended towards the surface, giving to it at last an inflammatory appearance, attended with induration, great hypertrophy, and the secretion of false membrane. The secretions from the tongue and salivary glands were tolerably copious in every case, and in some instances profuse; and there is but little doubt but that this circumstance exercised a favorable influence in mitigating, and perhaps in averting lesions in the cavity of the mouth that otherwise would have been of serious import. It may be stated: 1. That the disease uniformly affected some portion or other of the stomato-pharyngean mucous membrane; 2. that the local affection consisted of an active or sthenic hyperemia, seldom attended with ulceration or the secretion of coagulable lymph; 3. that the tongue, though not often, was nevertheless, at times found taking a conspicuous part in the pathology; 4. that in the instance to which we have alluded, where the tongue was the principal seat of the malady, it had no erysipelas upon its surface, the great hypertrophy and induration with which it was affected being apparent by the result of the morbid forces acting primarily upon its internal structure.

8. Having now noticed in a brief manner the lesions which may be regarded, in epidemic erysipelas, as primary, it remains for us to pay some attention to those of a secondary character, generally looked upon as *complications.* It should be remarked here, however, that it was very difficult to distinguish complications from the proper elements of the disease itself. Many cases, as has been previously stated, were ushered in by symptoms indicative of visceral disease. This was true, to some extent, of the viscera in all the splanchnic cavities. How, otherwise, than by supposing the viscera in these cavities to be primarily invaded, can be explained the cephalalgia, coma, and delirium, which in some instances followed the premonitory symptoms in such close succession, or the deep-seated burning pain and difficult respiration that attended other patients at the very commencement, or the violent pain and distention of the abdomen with which, almost universally, every puerperal case was affected? Circumstances of this kind go to sustain an opinion long since entertained by Hunter, and others, that erysipelas is a disease not peculiar to the skin, but liable to attack the mucous and serous membranes, or the viscera contained in any of the splanchnic cavities. Impressed, consequently with the belief, that much of what appeared to be consecutive, was really primary in its nature, it was nevertheless quite apparent, that in some instances the local organic disease supervened from circumstances purely accidental. Of this character was the disease of the brain which proved fatal in many instances where the face and scalp happened to be the seat of severe erysipelatous inflammation. About the same may be said of the pneumonitis and pleuritis, which succeeded to extensive erysipelatous inflammation and sloughing of the dermoid tissues covering the chest or superior extremities. Extension of the disease to the frontal and maxillary sinuses, although of rather uncommon occurrence, so far as my experience was concerned, was, nevertheless, from the serious character which it imparted to the disorder, when it did take place, of sufficient importance to entitle it to consideration. It was observed, in no instance, as being a primary element of the disorder, but always appeared as the result of extension from the cutaneous system covering the face and scalp. Indeed it took place only when the parts contiguous were the seat of violent erysipelatous inflammation. The discharges from the sinuses were fetid, and consisted of a thin bloody matter, mixed with pus, which denoted the presence of phlegmonous inflammation. Nothing was observed in relation to the condition of the alimentary canal which would favor the opinion of Abernathy and others, that in all cases of erysipelas the digestive apparatus is at fault. It is a fact that the digestive organs sometimes suffered; but as a general rule, this was not because they were the primary seat of the malady, but because of the close sympathetic relations they sustained to other portions of the body in a state of disease. In some instances there was diarrhœa, but it was very amenable to remedies, and as a consequence gave the physician no trouble. One case, also, is reported in this essay where the bowels became torpid after active disease of the brain supervened, and remained in this condition in spite of all appliances until the patient expired. Elementary writers on erysipelas speak of a complication of the disease attended with strong symptoms of disorder of the bilious system, constituting the *erysipelas phlegmonodes biliosum.* During the late epidemic I kept a look-out for complications of the liver, and although this organ was occasionally found within the circle of diseased actions, still I saw no instance of alterations, either functional or structural, sufficient to give character to the complaint. Bilious vomiting was of rare occurrence, and the same may be said of bilious diarrhœa, while the appearance of the skin, tunica albuginea, and tongue exhibited no more evidence of bilious disease than is usually found in synochus fever.

From the above inquiry into the different portions of the body affected by epidemic erysipelas, it may be stated in short, that the remote cause primarily displays its principal lesions:

1. Upon the lymphatic glands, giving rise to buboes and swellings of those glands on various parts of the body.

2. Upon the skin, in the different varieties of erysipelas, and other anamolous eruptions.

3. On the stomato-pharyngean mucous membrane, affecting with the most constancy and severity that portion of the membrane covering the tonsils, velum pendulum palati, and uvula.

4. On the tongue, this being of rather rare occurrence.

5. Upon the organs of generation, giving rise to an obscure form of puerperal disease.

The *complications* have been observed to arise from an extension of the inflammation:

1. To the brain and its appendages.
2. To the viscera of the thorax.
3. To those of the abdomen.
4. To the frontal and maxillary sinuses.

*Diagnosis.*—Well marked as has been epidemic erysipelas, wherever it has prevailed, and unique as it has appeared in most of its phenomena, whether of function or structure, there are, at the same time, some points of resemblance between it and other diseases that could not easily have escaped observation. The lesions found in the fauces resemble those found in the anginose variety of scarlet fever; and some practitioners, who have noticed this, have expressed an opinion, that the epidemic with which we have recently been visited is a combination of scarlatina with common sporadic erysipelas. The two diseases agree very well in the constancy with which the throat is affected, but there is a very wide difference in the character of the lesions. In epidemic erysipelas the anginose disturbance, as a general rule, is possessed of a higher grade of excitement, is of shorter duration, and is also unattended with those troublesome sequelæ which almost invariably follow scarlatina. In the puerperal state there is a very strong resemblance between erysipelas and puerperal fever, arising from other and very different causes. And so little is known in relation to the pathology of either of the diseases, that some danger of confounding them may reasonably be apprehended. In cases where the eruption, or the anginose affection is present, or where the disease happens during the prevalence of epidemic erysipelas, of course the difficulties in the diagnosis will be lessened. It will also be found that puerperal fever, arising from the virus of epidemic erysipelas, does not to any extent bear the use of the lancet, whereas in puerperal fever, arising from many other causes, there is great toleration of the loss of blood.

Some of the anatomical conditions observed in "*Anthracia pestus*," or plague, resemble in a striking manner, those found in epidemic erysipelas. Of this character are the buboes and erysipelatous eruptions, which occurred in the Aleppo plague of 1660–1–2, as described by Dr. Patrick Russel, physician at the time to the British Factory at Aleppo. Speaking of the London plague of 1665, Sydenham says: "In my opinion, the inflammation which the Latins call *ignis sacer*, and we *St. Anthony's fire*, or erysipelas, is very much like plague." Swan, Sydenham,s commentator, remarks: "The erysipelas and plague greatly resemble each other in the following particulars: 1. in their leading symptoms, viz: sudden shivering, loss of strength, violent pain in the head and back, vomiting, &c.; 2, in the expulsion of the malignant matter to the skin between the third and fourth day, with an abatement of the symptoms; 3. a tumor, redness and pain being first perceived near the groin, and thence descending to the feet; 4. in affecting the parotides when the head is threatened, and the glands of the arm-pit when the breast is endangered; 5. in the danger occasioned by the striking in of the morbid matter. In the *plague* of Athens, as described by Thucydides, we are told that, "the surface of the body was neither violently hot nor wan, but reddish, lined and covered over with an efflorescence of minute vesicles and ulcers." This author also says, that in some instances the disease commenced with an ulcerative process upon the head, and migrated over the entire frame, often fixing itself permanently on the sexual organs, the hands, or the feet. Writing upon the same subject, Sauvages remarks; "it commences with horror, burning heat, delirium, prostration of strength, vehement pain, pain of the back and head; in each, the burning matter of the disease breaks forth on the fourth day, on the axillary or inguinal glands, and spreads to the feet in the form of *ignis sacer;* in the glands it produces abscesses; in the extremities gangrene."

From these accounts of the phenomena which characterized plague, it would seem that the pathological anatomy of that disease, so far as the buboes and the affections of the skin are concerned, corresponds pretty well with epidemic erysipelas. By the writers which I have consulted on plague, no mention whatever is made of any anginose affection. Had this lesion, so constant in erysipelas, been mentioned in connection with plague, the points of analogy would have been sufficiently complete to justify the opinion that the two diseases, if not identical, are very closely related. It is indeed, extremely doubtful, whether a disease has ever made its appearance since the group of symptoms going to make up plague, was so rife in the land, that in more respects resembles that disease than does epidemic erysipelas. Incidentally, it may here be remarked, that various attempts have been made by modern writers to dissolve the group of phenomena usually found in plague, in order that they might be classed under other names, where their import would be better understood. Such efforts, too, have, to some extent been successful. But those who have advocated the propriety of dismissing plague from nosological systems, will find that epidemic erysipelas also presents an array of lesions, functional and structural, that are about as unique and as difficult of classification as those found in the plague, or even in Asiatic cholera.

*Prognosis.*—Much of what the physician is able to predict in regard to the issue of erysipelas depends upon the variety of the disease with which the patient is afflicted. The simple variety is usually attended with nothing from which danger need be apprehended. To a very considerable extent, the same is true of the lymphatic variety, or that form of the disease in which the lymphatic glands seem to be predominantly the seat of all of the more important alterations. Cases of this character, although at times very protracted, have usually a favorable termination; and, as far as my observation extends, no instance of this form has terminated fatally. When the skin on any portion of the body is the seat of an erysipelatous inflammation, the issue is of a character altogether less certain. The inflammation, owing to its insidious, wandering character, is liable at any time to seize upon parts vital, and thus destroy life. Less probability is there, however, of this event when the inflammation makes its appearance on the extremities, than when it is seated upon the head or face. In the latter situations, the physician, owing to the sudden and fatal turns which the disease often takes, can fore-tell almost nothing at all. True, as long as the integrity of the brain is preserved, no great danger need be apprehended. But who knows, in cases of this kind, from one moment to another what the consequences are going to be? Uncertain, to perhaps the same extent, are the results of those cases in which the inflammation first attacks some internal organ. In such instances the disease is, cæteris paribus, more liable to take life, than if it was the surface which was originally invaded. This, it is presumed, is in consequence of the forces of the organism becoming immediately prostrated from the stroke made upon the nervous system. When the tongue is implicated, so as to give rise to dysphagia and dysphonia, the patient's life is in great jeopardy. As before remarked, my acquaintance with disease of the tongue has been limited, yet if what I have seen be a fair sample of the degree of danger to be apprehended, when this organ is the principal seat of the the trouble, nothing can be predicted in favor of recovery. In the case reported in this paper it was a number of days before apprehensions ceased that the patient would die of strangulation. The puerperal variety is less equivocal in its results than any form of the disease yet considered. The morbid forces in this variety display a degree of malignity that may generally be interpreted to the prejudice of the patient's recovery. Out of all the cases which have occurred in the Miami valley, or in the districts bordering on this valley, not more, to say the least, than the twentieth one has recovered. This is a degree of mortality, that not only cuts off all hope on the part of the patient, but leaves the physician without any other alternative than the humiliating one of making an unfavorable prognosis in all cases. From what we have witnessed in regard to the age of persons most endangered by an attack of erysipelas, it seems very probable that less can be predicted in favor of old or aged persons than of any other class. In such, the shock made upon the nervous centres, when the disease is even comparatively mild, is frequently sufficient to take life. I attended to a lady in her seventy-fourth year who seemed to me to be afflicted with the mild variety, and of course my prognosis at the commencement was rather favorable. But, without being characterized by either buboes or erysipelatous inflammation, or indeed by any phenomena of a grave character, other than occasional incoherence and delirium, the latter of which took place only in the last stage, the case went on to a fatal issue. In other instances, where the organism was pretty well worn, the malady exhibited a degree of malignity from which persons in the prime of life were exempt. Young children that contract the disorder from puerperal mothers are very apt to die. Indeed, the recoveries have been so rare, that cases of this kind are looked upon as being necessarily fatal.

*Course and Termination.*—There is a certain cycle of changes through which many diseases will run, as for example those of the skin, and from which they cannot be moved by any means known to the art of medicine. Eminently, this is true of erysipelas in all its varieties. But while a certain portion of time is required, the length of time varies according to the form with which the patient is afflicted. When an individual is only affected with a chill, followed by a moderate fever, little soreness of throat, and slight swelling of the lymphatic glands, the disease requires about five or six days to run its course. Cases attended with an erysipelatous eruption last longer. Commonly it is not until about the fourth day that the eruption is developed, and after this from four to six days are required before the eruption subsides, making the course of this variety to be eight or ten days. It should be remarked here, however, that the form of the disease, of which we are now speaking, when it runs into phlegmonous erysipelas, becomes at times very protracted, being commensurate with the continuance of the local disease. That form of the malady which displays its morbid forces principally upon the axillary and inguinal glands, is less amenable to any definite length of time than any of the varieties considered. It often happens that patients have to pass through a process of suppuration and ulceration, exceedingly indolent in its character, but sufficient to keep the principal symptoms of the disorder upon the patient for weeks.

The termination, in almost all instances where the disease proves fatal, is attended with more or less disturbance of the nervous centres. Many of the fatal cases were characterized by erysipelatous inflammation of parts contiguous to the brain, and of course this circumstance will explain the cerebral symptoms during the last moments. But I believe most of the puerperal women, as well as their children, were affected with violent delirium for some time preceding dissolution. In some instances the termination, I have learned from other practitioners, is by suffocation. These were attended with very decided disease of the respiratory apparatus from the beginning, and not unfrequently with the signs of inflammation of the diaphragm. Few cases were marked in the last stages with diarrhœa, or any other symptoms denoting much previous disease of the mucous surface of the bowels.

### TREATMENT, GENERAL AND LOCAL.

As is frequently the case, nothing can be proposed in reference to the treatment of epidemic erysipelas, either general or local, that can be said to be applicable to all cases. Varied as it must be, according to the form of the disease with which the patient is afflicted, it must also be more or less modified by diathesis, habit, and age. No one, who has had any experience in the malady, has failed to notice, that, in all its varieties, it seems to be more or less connected with an exalted condition of the principal functions of life. The fever present in the commencement is generally of the synochus grade, attended with a full, and in many instances a very frequent pulse. Blood drawn, when not covered with a buffy coat, presents always that firm, rich appearance which it usually assumes before fibrin is developed on its surface. I have seen no instance of a dissolved putrid appearance of the blood, such as is witnessed in the blood of persons laboring under decided adynamic diseases. Everything indeed, either functional or structural, evinces that the prevailing diathesis, during the period of incubation, and after the malady is completely formed, is phlogistic. From circumstances of this character, the propriety of antiphlogistic remedies could not be called in question. But as the different varieties require more or less difference in the treatment, it may not be out of place to give a few of the most important remedies a special consideration.

*Bloodletting* is applicable to all the forms of the malady.

In the simple variety its use, though not so decidedly indicated, is nevertheless very obvious. Pretty uniformly it has been my practice to let blood at the very onset of the disease, and this to an extent sufficient to make a decided impression upon the system. I have found that this practice in many cases subdues the more urgent symptoms at once, and places the patient out of danger. But, even in cases, where this salutary result is not obtained, its effects, in mitigating the symptoms, and bringing the disease within the reach of other remedies are very apparent. The evidence has been very decided, also, that vigorous depletion at the commencement when the phenomena are at all portending, is the only means upon which reliance can be placed, that seem calculated to prevent erysipelatous eruptions and visceral inflammations. In cases where the affection, in the first stages, is attended with deep-seated burning pain in the chest or abdomen, abstractions of blood *ad deliquium*, is the remedy upon which I mostly rely; and, if the first bleeding does not remove the pain, it should be repeated. In that form of the disease in which the skin is implicated, consisting of violent erysipelatous inflammation, bleeding is indispensable. Here, in many instances, before the eruption makes its appearance, the action of the heart and arteries is so vehement that the patient is thrown into coma and delirium. I saw a young man, of good constitution, who had been seized with the disease only a few hours previous to my visit. The pulse was 130; the respiration very much hurried and laborious; the tongue dry and red; the breath hot and fœtid, and the functions of the brain so much disturbed that he was delirious. He was bled until he became sick and vomited. This made such an impression upon the disease that all the more urgent symptoms were immediately allayed, and under the use of cathartics he was in a few days restored to health. Assured as I was, that erysipelatous inflammation would have been developed in this case, had not prompt and vigorous blood-letting been adopted; I felt confident that it was the *only*

means that could have been successfully used. After the eruption makes its appearance, and while it is in a state of migration, sanguineous evacuations, although not so decidedly salutary as at earlier periods, are still necessary to moderate the circulation, and in this way prevent the eruption from extending to parts that endanger life. It should now be remarked, that while bloodletting is applicable to the first stages of all the varieties of the complaint, and is the chief means from which the most salutary results may always be expected, there is at the same time a point beyond which it is not safe to proceed. When the vehemence of the inflammatory symptoms is subdued, and the other indications, of which we have just spoken, are fulfilled, and the disease still fails to yield, further depletion may make the pulse more frequent and irritable, and precipitate the patient into a typhoid condition, always prejudicial to recovery and sometimes fatal. Hence, all the practitioners of experience, with whom I have conversed, agree as a general rule upon the propriety of vigorous depletion at the commencement, and think this to be the period to which its usefulness is mostly confined. As long as we can use depletion to subdue the disease without interfering too much with the integrity of the vital forces, it should not be omitted. But when this cannot be done, such measúres should be suspended, and other means instituted less calculated to jeopardize the resources of the system. The vital influence of the blood, all know, not only does much in resisting the inroads of the disease, but it is indispensable to recovery, and for these reasons should not be carelessly wasted. Besides the caution that it is necessary to observe in the different stages relative to the extent to which the use of the lancet should be carried, there are other circumstances with which all are more or less familiar, that go to qualify measures of depletion. Old persons in many instances will not bear venesection at all. Nor can it be practised with any certainty in habits corrupted by intemperence, or broken down by vice of any kind.

Again, in a diathesis decidedly phthisical or scrofulous, connected with disease of some of the great functions of life, abstractions from the vital current are often prejudicial, and at best are of questionable propriety. With these remarks on the utility of bloodletting, and the circumstances by which its use should be qualified, I proceed to a consideration of

*Cathartics.*—Medicines which induce alvine evacuations are of various kinds, as it regards the effect they produce upon the mucous surface of the bowels, and have several modes of operation. From some attention paid to these peculiarities during the prevalence of erysipelas, I have found that some cathartics, as a general rule, are of much more service in fulfilling the indications which the disease presents for this class of remedies, than others. Those that excite purging, and at the same time exercise an antiphlogistic influence over the system, giving rise to copious liquid evacuations, are in the majority of cases most applicable. After the use of the lancet it has been my practice to give a combination of cream of tartar and jalap in doses sufficient to produce free catharsis. These means are often of themselves sufficient to subdue the complaint. It happens occasionally that the disease is protracted with complications, during which it is necessary to keep up regular alvine evacuatons for some time. For this the Seidlitz powders answer a good purpose. I have used mercurials and some of the more stimulating cathartics; and sometimes in old persons or in those of bad habits with advantage. In the great majority of cases, however, those of a saline hydragogue character are not only indicated, but will display the best effects.

*Emetics.*—Practitioners of medicine with whom I have conversed, generally bear testimony to the usefulness of emetics; and so far as my own experience is concerned it is decidedly in their favor. Their value is mostly observable in giving to the fluids of the system a centrifugal direction by which congestions upon the deep-seated organs are obviated, and also in preventing defluxions about the throat. There is a salutary influence which the process of emesis exerts, by establishing something like an equilibrium, and by driving the fluids towards the surface, that although of much importance in many diseases, is seen in few with more decided benefit than in erysipelas. About the same may be said relative to the control which they possess over affections of the throat. All know, that in angina maligna emetics not only cleanse the fauces, but exercise a curative influence over the parts locally diseased. They do the same thing in erysipelas when the throat and tongue are much affected. When the tongue is the seat of severe disease, the breathing at times becomes laborious, the deglutition difficult, and the faculty of speech almost entirely suspended. Under such circumstances emetics are invaluable, and are almost the only means we possess capable of remedying these grave phenomena. As it regards the particular emetic best calculated to fulfil the indications, our experience is too limited to speak with any degree of certainty. Towards ipecacuanha, administered in a solution of common salt, I have considerable partiality. This makes an emetic sufficiently active for all the purposes for which it is designed, while the salt of which it is in part composed exerts a very favorable influence over the fœtid breath, and the viscid irritating accumulations that collect in the fauces.

*Tonics.*—Of the use of strengthening medicines I know but little. The epidemic with which our region was visited very seldom indicated that they were admissible. It was, as has been repeatedly mentioned, attended in the first stages with a group of symptoms decidedly inflammatory; and even in cases where the disease became protracted, there were generally visceral affections, or inflammation of the lymphatic glands, that made medicines calculated to give tone or force to the circulation, of questionable propriety. In broken down constitutions, or in cases laboring under the influence of old age, or where, from protracted suppuration or sloughing, the powers of the constitution are exhausted, of course such means are occasionally indicated, and will prove decidedly beneficial. These conditions I have seldom encountered during the prevalence of the late epidemic. Prof. Mussey, however, informed me that they were of frequent occurrence among the patients in the surgical wards of the Cincinnati Hospital, and that stimulants and tonics had to be pretty liberally used. From all that I can learn of the prevalence of epidemic erysipelas in hospitals, it seems to be connected with a lower grade of fever, and symptoms more adynamic, than is found in the same disease when it prevails in the country. This circumstance, if correct, explains the frequent necessity, in these institutions, of resorting to means in the treatment that produce a strengthening effect, and have a tendency to assist in economising the resources of the system.

To sum up what I have to say on the general treatment I may state:

1. That the prevailing diathesis in country situations, at any rate, is inflammatory.

2. That antiphlogistic remedies of a decided character are not only indicated, but as a general rule may be successfully adopted in practice.

3. That bloodletting is most applicable to the first stages, and is usually attended with the most salutary results.

4. That the circumstances attending a case which contra-indicate the use of antiphlogistic remedies depend upon some fault in the constitution, habit or age of the patient, and not upon the nature of the pervading diathesis.

*Topical Remedies.*—Various are the opinions of medical and chirurgical writers on the propriety of topical applications to the parts in a state of erysipelatous inflammation. This want of unanimity I have found also to exist, to a very considerable extent, among practitioners of much experience in the late epidemic. They speak in very ambiguous terms relative to the usefulness of this class of remedies. In many instances they could see no benefit whatever arising from their use. Indeed a large proportion of the profession at the present time, at the head of whom stands Dessault, regard them as being decidedly unnecessary, if not injurious. Objections to their use are mainly founded upon the position—"that erysipelas is an idiopathic disease, giving rise to eruptions on the skin that bear about the same pathological relations to the general disease, which is witnessed in the proper exanthemata between the general disease and the eruptions upon the surface; and second, that local applications having a tendency to destroy the eruptions upon the surface, if successful, can do no good until the general disease is subdued, and even then may prove prejudicial by driving the disease from the skin on some organ more calculated to endanger life." Pertinent as these objections are, their principal value, it is likely consists in inspiring the physician with caution in ascertaining the circumstances by which the use of local remedies should be regulated, rather than in proscribing them altogether from the treatment. While the disease is characterized with a high grade of vascular excitement they will not only fail in affording any relief, but may repel the inflammation from the surface, and thus prove highly injurious. In cases too where the local affection occupies a great extent of the surface, but little good can arise from applications that exercise more than a mere soothing influence. After the general disease is subdued, and there yet remains a tendency in the local affection to extend itself to contiguous parts, local applications are at times attended with excellent results. It often happens also that after the more urgent symptoms have subsided there is still present an erysipelatous inflammation that serves as a focus from which proceeds a morbid influence that keeps the patient in a suffering, irritable condition. Local applications here are serviceable. Concerning the *special* remedy most proper there is a diversity of opinion.

*Nitrate of Silver* has been before the profession for some time, and has sustained with many the character of a very efficient agent. It is used in all stages of the eruption, as well at the time that it first makes its appearance, as when it passes on to a high degree of inflammation, or at the period of sloughing and ulceration. From this indiscriminate application of the remedy it would be reasonable to expect that confidence in its virtues would become impaired. The therapeutic effects of nitrate of silver, as displayed in other diseases, warrant us in concluding that it is not as applicable to acute as chronic inflammations, and as a matter of course, in that kind of alteration present in the parts during the period of suppuration, as well as a little anterior, its usefulness is most obvious. Here something is needed to alter the action of the parts and excite granulation. I am aware that the medicine is now used in the various hospitals of the country for the purpose, as much as anything else, of arresting the erysipelatous inflammation. But its powers in this respect are comparatively limited. Velpeau regards it as being almost useless.

*Corrosive Sublimate.*—Some very flattering accounts have been published concerning the efficacy of this drug. I have used it to some extent in the acute stage of the eruption, and really I was disappointed. It seemed to aggravate the intense burning pain of the parts, without producing any curative effect whatever. The substance seems to me to be too severe and irritating for an acute inflammation, such as we have in erysipelas; and indeed, I think the article altogether more applicable to some of those chronic eruptions classed by Willan under the order squamosa than to erysipelas.

Dissatisfied with the usual remedies employed in erysipelas, M. Velpeau was induced to give *sulphate of iron* a fair trial. The idea that it would be beneficial suggested itself to him from a consideration of the modifications which some of its preparations are calculated to induce in the blood; and he states that in twenty-four cases in which he employed the article the most marked and rapid influence was exerted over the progress of the eruption. From twenty-four to forty-eight hours was the time required to complete a cure. The manner in which Velpeau used the article was in solution, an ounce of the sulphate of iron to a pint of water; or in the form of an ointment, containing two drachms to the ounce of lard. There is one singular circumstance connected with Velpeau's use of iron. It seemed to him to cure the eruption but would not prevent it from spreading, if even the parts were previously smeared over with the ointment. This leaves some room for doubting the efficacy of the remedy. It may be, that the distinguished Parisian author confounded the spontaneous subsidence of the eruption with the effects of his medicine. Or is the medicine simply curative and not preventive? Drs. Vantuyl, Jewett, and Steel, of Dayton, all used the sulphate of iron as an external application in the late epidemic, but neither of them could report anything in its favor. The authority, however, of Velpeau is a sufficient recommendation to induce practitioners to give the article a fair trial.

*Flying Blisters*, as they are sometimes called, have had considerable reputation in France and in this country, in putting a stop to the extension of the inflammation. Dupuytren speaks very favorably of this practice, and I believe it has enjoyed the confidence of some of the most distinguished medical men in the United States. The claims of this remedy, during the prevalence of erysipelas among us, were not overlooked. I have scarcely conversed with a practitioner who has not given it a trial; and its powers of putting a stop to the erysipelatous inflammation are exceedingly limited. In a case where I used it, the inflammation had first made its appearance on the end of the nose, and afforded a very good opportunity to test the efficacy of the remedy. Strips of blistering-plaster were applied all around the inflammaton upon the sound skin so as completely to circumscribe the part affected. They, however, did no good, the inflammation extended in every direction without seeming to have the appearance of even being checked. I have no experience in the application of blisters to the disk of the inflammation, but confess that my prepossessions concerning the usefulness of such practice are by no means strong.

*Compression by Bandages*, as recommended by Bretonneau and Velpeau, is spoken of favorably, when the inflammation is situated on the extremities. Of course the remedy cannot be used about the face or neck. One of our American authors, I allude to the late Dr. Eberle, says that he had "a satisfactory illustration of the usefulness of this measure." Dr. Eberle only gives one case in which he tried it, but from the close observation which it was his custom to make, the remedy, in my opinion, is entitled to consideration.

*Iodine* lately has been brought forward as a remedy of undoubted virtue in many disorders of the skin, and, among the rest in erysipelas. Pereira says that in erysipelas he has seen it decidedly beneficial. Testimony equally strong from writers in Europe and America might be adduced in confirmation of what is said by Pereira. In truth, the reputation of this substance has been built up so rapidly, and apparently on such a firm foundation, that it seems almost like presumption to assail it. But facts, whether *pro* or *con* must have their influence. In an essay published from his pen, "*On the topical application of iodine and its compounds*," in Braithwaite's Retrospect, No. VI, James J. Ross, M.D., says: "Some writers have lauded the local application of the tincture of iodine in erysipelas as if its use were really a specific for the disease, and rendered all other treatment superfluous. I have used it in several cases, but in any instance of pure phlegmonous erysipelas, I have never seen it equal to the subduction of the disease however early employed." To a humiliating extent the experience of Dr. Ross has been confirmed by the practitioners of our own country, extensively engaged in the treatment of epidemic erysipelas. The medicine has been generally used; and that too with the confidence in its effects which its previous reputation was calculated to inspire; and while some incline to a favorable opinion of its merits, others there are, and that too of a numerous class, who regard it as being comparatively useless. My own experience with the drug is not great; but so far as I have witnessed its effects I can report nothing special in its favor. Applied to a raw sloughing surface, patients complain very much of the irritation it produces; and this *cæteris paribus*, would be an objection in many cases to its use. The attempts which I have made with the article to subdue the eruption, in the acute stage, have not been at all successful.

I might here notice other medicines, such as *lime-water*, the *black wash*, *chloride of lime*, and unguents of various kinds, some of which are as favorably spoken of, as any of the topical applications to which we have alluded, but my remarks have been as much extended as perhaps the real usefulness of the whole class would seem to demand; and I shall therefore proceed to consider a measure of a less doubtful character. I allude to

*Incisions.*—This remedy since the time that it was recommended by Lawrence, has shared largely of the confidence of a great number of medical men, whose opportunities of testing its usefulness have been ample, and in whose judgment reliance may well be placed. Lawrence asserts that *incisions* are quickly and almost instantaneously followed by relief and cessation of the pain and tension; and I am moreover assured that this alleviation of the local suffering is accompanied by a corresponding interruption of the inflammation, whether it be in the stage of effusion or in the more advanced period of suppuration. In the advanced stage the incisions limit the extent of suppuration and gangrene, and by affording a ready outlet for matter and sloughs, facilitate the commencement and progress of granulation. *

* Dictionary of Practical Surgery. By Sam. Cooper. From Med. and Chirurgical Trans. Vol. 14, page 67, &c.

Sanguine as these views are concerning the efficacy of this measure, they have to a very considerable extent been confirmed wherever the remedy has been extensively used. As it regards the manner in which the incisions should be made there has been quite a diversity of opinion expressed. Some recommend mere punctures, others limited incisions, while not a few think most favorably of long gashes. Without doubt the propriety or impropriety of either of these modes depends upon circumstances. When the affection on the skin is superficial and not attended with any great pain or tension, puncturing with a fine lancet at numerous points as recommended by Sir R. Dobson will do; but in severe cases where the tension and suffering evidently arise from an over-distended condition of the bloodvessels, incisions of sufficient depth to deplete the parts freely, and give exit to the confined blood and serum are evidently required; and when the extremities are extensively affected, long incisions are most serviceable. Hence it is that no specific directions relative to the manner in which the incisions should be made are of universal application. In a case where the principal inflammation was situated on the face and to some extent on the scalp, I made incisions to the number of nine or ten, and of depth and length sufficient to deplete the parts of blood and serum. There was at the time the incisions were made no collections of pus beneath the skin, and as a consequence it was not necessary that the skin should be completely divided. Immediately after the incisions were made my patient expressed great relief, and it was but a short time until convalescence was complete. My experience in the usefulness of this measure has not been extensive. Nor do I think that it has generally been resorted to by practitioners in the treatment of epidemic erysipelas. One thing, however, is very certain, wherever it has been tried its control over the disease is invariably acknowledged, and it is at once admitted to take precedence of all other appliances.

From this brief inquiry into the therapeutic effects of topical remedies, the following conclusions are apparent:

1. A great number of medicines have been used, and there is scarcely one but what has received the sanction of somebody or other in the profession.

2. During the prevalence of the late epidemic, few, if any of the medicines reputed to be efficacious in curing the eruption, or in preventing it from spreading, fulfilled the expectations of the practioners.

3. The practice of incisions, since first proposed, has been steadily gaining confidence, and is now, without any doubt, the most available means known to the science of medicine.

### TREATMENT OF PUERPERAL CASES.

Confessedly my object in giving to this subject a special consideration, is not because anything can be proposed in the shape of a remedy, but that the attention of the profession should be called to the humiliating fact, that the treatment of such cases, as far as can be learned, has been unsuccessful. The physicians of Dayton and Centreville candidly informed me that all their efforts proved fruitless. This is the testimony also of those who have given us accounts of the prevalence of the disease in the lying-in wards of hospitals. In all cases, with scarcely an exception, it runs its course and terminates fatally. Depletion has been tried, but, in the general way, the pulse gets frequent, irritable, and small under the use of the lancet. The same is true of other means decidedly antiphlogistic. Well, as it regards stimulants, they appear to be strongly contraindicated; and on this account, so far as I have learned, have been withheld. The disease seems to be both inflammatory and rapidly prostrating in its character; and in consequence of these circum stances presents a complication of phenomena, for the relief of which it is not easy to prescribe.

In the Transactions of the College of Physicians of Philadelphia, Dr. Huston reports one recovery in the lying-in ward of the Philadelphia Hospital, which followed the use of a full dose of opium, (two grains) with calomel, sinapisms to the feet, carbonate of ammonia, &c. Dr. Huston was induced to try this practice from the fact, that the cases which had been treated by venesection and other means of a decidedly antiphlogistic character all terminated fatally. I am not aware that calomel and opium in conjunction with ammonia, have been much employed by practitioners of the western country in the late epidemic. One thing, however, is evident, that it is our imperative duty, after finding what seems to be obviously indicated fail entirely, to resort to other means, until there shall be something found which will remove this *opprobrium medicorum* from our profession.

June 25th, 1845.

[*Note.*—In the Louisville Marine Hospital, where from first to last a good deal has been seen of epidemic erysipelas, it has been the experience of the physicians, we believe, that bloodletting is a dangerous remedy in that complaint. It has been stated to us that in every case, in which the patient was subjected to a loss of blood, the issue was fatal. It would seem that this disease assumes a new character in the atmosphere of a hospital. Y.]

